# Age at Menarche and Incidence of Diabetes: A Prospective Study of 300,000 Women in China

**DOI:** 10.1093/aje/kwx219

**Published:** 2017-06-09

**Authors:** Ling Yang, Liming Li, Sanne A E Peters, Robert Clarke, Yu Guo, Yiping Chen, Zheng Bian, Paul Sherliker, Jiyuan Yin, Zhenzhu Tang, Chunmei Wang, Xiaohuan Wang, Libo Zhang, Mark Woodward, Zhengming Chen

**Affiliations:** 1 Medical Research Council Population Health Research Unit, Nuffield Department of Population Health, University of Oxford, Oxford, United Kingdom; 2 Clinical Trial Service Unit and Epidemiological Studies Unit; 3 Chinese Academy of Medical Sciences, Dong Cheng District, Beijing, China; 4 Department of Public Health, School of Public Health, Peking University, Beijing, China; 5 The George Institute for Global Health, University of Oxford, Oxford, United Kingdom; 6 Heilongjiang Center for Disease Control and Prevention; 7 Liuzhou CDC, Liuzhou, Guangxi, China; 8 Tongxiang CDC NCDs Prevention and Control Department, Tongxiang, Zhejiang, China; 9 Hainan CDC NCDs Prevention and Control Department, Haikou, Hainan, China; 10 Liuyang CDC NCDs Prevention and Control Department, Liuyang, Hunan, China; 11 The George Institute for Global Health, University of Sydney, Australia

**Keywords:** China, diabetes mellitus, menarche, prospective studies, women

## Abstract

Previous studies of predominantly Western populations have reported inconsistent associations between age at menarche and risk of diabetes. We examined this relationship among Chinese women, who generally experience menarche at a later age than Western women. In 2004–2008, China Kadoorie Biobank recruited 302,632 women aged 30–79 years from 10 areas across China, and recorded 5,391 incident cases of diabetes during 7 years of follow-up among 270,345 women without baseline diabetes, cardiovascular disease or cancer. Cox regression models yielded adjusted hazard ratios for incident diabetes associated with age at menarche. Overall, the mean age at menarche was 15.4 years, and decreased across successive generations. Age at menarche was linearly and inversely associated with incident diabetes, with adjusted hazard ratio of 0.96 (95% confidence interval (CI): 0.94, 0.97) per year delay. Hazard ratios were greater in younger generations (for women born in the 1960s–1970s, hazard ratio (HR) = 0.93, 95% CI: 0.90, 0.97; for women born in the 1950s, HR = 0.95, 95% CI: 0.93, 0.98; and for women born in the 1920s–1940s, HR = 0.97, 95% CI: 0.95, 0.99). Further adjustment for adulthood body mass index significantly attenuated the association (HR = 0.99, 95% CI: 0.97, 1.00), especially among those born before 1950 (HR = 1.00, 95% CI: 0.97, 1.02). Much of the inverse association between age at menarche and incident diabetes was mediated through increased adiposity associated with early menarche, especially in older generations.

Diabetes currently affects over 400 million people worldwide and causes a substantial burden of premature death and disability (1). In China, there has been a rapid increase in the prevalence of diabetes over the last three decades, occurring in an estimated 100 million adults (12.1% men and 11.0% women), even though mean body mass index (BMI) has remained much lower compared with Western populations (2, 3). Secular trends in diabetes prevalence have coincided with a decline in the average age at menarche among women in China (3, 4). There is ample evidence from Western populations that early menarche is associated with excessive adiposity in adulthood (5), as well as with elevated levels of blood glucose or insulin resistance, independent of adiposity (6, 7). However, previous evidence for the association of age at menarche with risk of diabetes has been inconsistent. Some studies report null or positive associations, while others show inverse associations, mainly with regard to prevalent rather than incident diabetes. Most previous studies have been conducted in Western populations in which women generally have higher BMI and earlier age at menarche, and tended to have small sample sizes with insufficient control of confounding factors. Moreover, substantial uncertainty persists as to whether the association between age at menarche and diabetes may be mediated entirely by adiposity (8–11). We examined the associations of age at menarche with diabetes in a large 7-year prospective cohort study of the China Kadoorie Biobank, which consisted of 300,000 women who were born between the 1920s and 1970s.

## METHODS

### Baseline survey

Details of the China Kadoorie Biobank study population have been reported previously (12). Briefly, the baseline survey was conducted from 2004 to 2008 in 10 areas across China. The regional study sites were carefully selected to retain geographic and social diversity, as well as to maximize difference in disease rates and risk exposure in order to approximate nationally representative samples. Data on general demographic and socioeconomic status, body weight at age 25, dietary and other lifestyle habits (e.g., smoking, alcohol drinking and physical activity level) were collected using an interviewer-administered laptop-based questionnaire. Questions on medical history determined whether participants had ever been diagnosed with a range of chronic diseases (e.g., diabetes, heart disease, stroke, and cancer) by a physician. Women were asked about their reproductive history, including age at menarche, parity, age at birth and breastfeeding duration for each live birth, and menopausal status, as well as history of oral contraceptive (OC) use. All participants provided written consent. International, national, and local ethics approval was obtained.

Measurements of blood pressure, random plasma glucose (RPG) and anthropometry were done by trained health workers using standardized protocol and procedures. Blood pressure was measured at least twice after participants had remained at rest in a seated position for at least 5 minutes, using a UA-779 digital monitor (13). Anthropometric measurements were taken while participants were wearing light clothes and no shoes. Waist circumference (WC) was measured midway between the lowest rib and the iliac crest, using a soft non-stretchable tape. Body weight was measured using a bioelectrical impedance device (TANITA-TBF-300GS; Tanita Corp, Tokyo, Japan). Standing height was measured to the nearest 0.1 centimeter, using a stadiometer. BMI was calculated as weight in kilograms divided by the square of standing height in meters (14). RPG levels were measured using the Johnson and Johnson SureStep Plus System (LifeScan, Milipitas, California, USA). Those without previously-diagnosed diabetes but with an on-site RPG level of 7.8–11.1 mmol/L, were invited to undergo fasting plasma glucose testing the following day. Screen-detected diabetes was defined as the absence of self-reported diabetes, together with the presence of measured RPG level of 7.0 mmol/L or greater with more than 8 hours since last food intake, or measured RPG level of 11.1 mmol/L or greater with less than 8 hours since last food intake, or fasting plasma glucose level of 7.0 mmol/L or greater on subsequent testing (15). All devices were regularly calibrated to ensure the consistency of measurements.

### Follow-up for mortality and morbidity

Participants were followed-up for cause-specific morbidity and mortality through linkage with regional disease and death registers. All hospitalized events were monitored through electronic data linkage with the nationwide health insurance system, which has almost universal coverage and captures episodes of new-onset diabetes for both outpatients and hospitalized patients. Causes of death were sought chiefly from official death certificates, and if necessary, supplemented by reviewing medical records or undertaking verbal autopsy. Verbal autopsy is a World Health Organization standard tool to determine probable causes of death for people who died without any medical attention, or for those with reported ill-defined or unknown causes of death (16). To minimize loss to follow-up, annual contact was made with many participants, and annual visits were made to local communities (12). Fatal and non-fatal events were coded according to the *International Classification of Diseases, 10th Revision*, and blinded to baseline information. The start of follow-up was defined as the date of enrollment into the baseline survey for each participant. Person-years were calculated until the date of diabetes diagnosis at the hospital, date of death, loss to follow-up, or the date of study termination (December 31, 2013), whichever occurred first. For new onset of diabetes, the validity of diagnosis was adjudicated in a random sample of 831 reported cases, and involved careful review of hospital records. Overall, 98.6% diagnoses of diabetes were confirmed.

### Statistical methods

For the present study, 32,287 participants were excluded because of missing, inconsistent, or implausible values of reproductive factors (*n* = 1,980), prior history of cardiovascular disease or cancer (*n* = 12,485), and self-reported diabetes or screen-detected diabetes (*n* = 17,822) at baseline (17). Cox proportional hazards models were used to estimate hazard ratios, with age at menarche as the exposure variable, which used age at menarche of 13 years as the reference group and diabetes as the outcome (Web Table 1, available at https://academic.oup.com/aje).

Adjustments for confounding factors were conducted in 4 sequential models. Model 1 involved stratification by 5-year groups of age at risk, region (10 areas) and the highest level of attained education (no formal education, primary, secondary, tertiary school or higher education (college/university)). Model 2 was further adjusted for lifestyle factors such as smoking (never, occasional, ex-regular, current regular), alcohol drinking (never, occasional, ex-regular, reduced intake, weekly intake), and physical activity level (metabolic equivalent-hours/day) (18). Model 3 was adjusted for other reproductive factors, including menopause status, parity, age at first birth, breastfeeding duration, and OC use. Model 4 was additionally adjusted for measured blood pressure at baseline, which we took as our primary analysis. On the basis of model 4, we examined whether associations may differ across different population subgroups defined by region, education, birth cohort, menopausal status, smoking status, alcohol drinking status and hypertension history, which are major risk factors of diabetes. To assess possible mediation by adiposity, additional analyses were conducted by separately adding baseline BMI, WC, or both BMI and WC to model 4. To examine the effect of early adulthood adiposity on the association, BMI at age 25 (based on reported weight at age 25 and height measured at baseline) was also added to model 4 without including adult adiposity. Sensitivity analyses were conducted among women who had never smoked, drank alcohol or used OCs. The 95% confidence intervals for each log hazard ratio were estimated using the “floating absolute risk” method, which facilitates statistical comparisons between different categories of age at menarche, rather than only pair-wise comparisons between one arbitrarily chosen reference group and each of the other categories (19). To correct for regression dilution bias related to reporting error in age at menarche, hazard ratios in the groups determined at baseline were plotted against the usual age at menarche (i.e., the mean value of age at menarche in that group at the 2008 resurvey) (20, 21). Analyses were performed using SAS, version 9.3 (SAS Institute, Inc., Cary, North Carolina) and R, version 3.0.1 (R Foundation for Statistical Computing, Vienna, Austria).

## RESULTS

Among 270,345 women included, the mean age at recruitment was 50.1 (standard deviation (SD), 10.3) years, mean BMI was 23.7 (SD, 3.4), WC was 78.5 (SD, 9.3) cm, RPG was 5.7 (SD, 1.1) mmol/L, and 43% were urban residents (Table 1). The majority of women were not current smokers (98%), or regular alcohol drinkers (98%), or users of OCs (90%). Overall the mean age at menarche was 15.4 (SD, 1.9) years, which decreased from 16.2 (SD, 2.0) years to 15.6 (SD, 1.9) years to 14.7 (SD, 1.7) years among women born before 1950, during the 1950s, and after 1959, respectively. On average, women with younger age at menarche were much younger at baseline, more likely to reside in urban areas, be better educated, and have a lower average blood pressure. After adjustment for other lifestyle factors, age at menarche was not associated with RPG but was linearly inversely associated with adult adiposity. Each year of earlier onset of menarche was associated with a 0.35 cm higher baseline WC, 0.18 higher baseline BMI, and 0.07 higher BMI at age 25 (all *P* values < 0.001) (Web Figure 1). Nearly all women had given birth (99%) and breastfed their children (97%), with a mean age at first birth of 23.4 (SD, 3.1) years. Compared with women with later menarche, women with earlier menarche tended to have fewer children, slightly later age at first birth, shorter duration of breastfeeding, earlier age at menopause, and longer total reproductive years (Table 1).

**Table 1.** Baseline Characteristics of Female Participants, China Kadoorie Biobank Study, China, 2004–2008

Characteristic	Overall		Age at Menarche, years													
			≤12		13		14		15		16		17		≥18	
	(*n* = 270,345)		(*n* = 14,685)		(*n* = 31,358)		(*n* = 45,346)		(*n* = 51,046)		(*n* = 50,089)		(*n* = 38,178)		(*n* = 39,643)	
	Mean (SD)	%	Mean (SD)	%	Mean (SD)	%	Mean (SD)	%	Mean (SD)	%	Mean (SD)	%	Mean (SD)	%	Mean (SD)	%
Age, years	50.1 (10.3)		45.9 (9.4)		46.4 (9.6)		47.0 (9.9)		49.1 (10.2)		50.9 (10.0)		52.9 (9.8)		55.6 (8.9)	
Lifestyle factors and physical measurements																
Urban resident		42.9		52.3		51.8		45.2		41.8		40.8		40.0		35.4
No formal school		25.0		22.8		21.5		21.8		23.8		25.1		26.6		30.5
Current regular smoker		2.2		2.3		2.1		2.2		2.2		2.3		2.3		2.3
Weekly regular drinker		2.1		2.4		2.1		2.0		1.9		2.1		2.2		2.5
Physical activity level, MET-hours/day	21.1 (12.8)		21.7 (12.7)		21.9 (12.9)		22.0 (12.9)		21.8 (13.2)		21.2 (13.0)		20.4 (12.8)		19.1 (12.0)	
Random plasma glucose, mmol/L	5.7 (1.1)		5.7 (1.1)		5.7 (1.1)		5.7 (1.1)		5.7 (1.1)		5.7 (1.1)		5.8 (1.1)		5.8 (1.1)	
Systolic blood pressure, mmHg	128.6 (21.5)		125.4 (20.6)		125.4 (20.2)		126.3 (20.8)		128.5 (21.3)		129.4 (21.5)		130.7 (21.8)		132.4 (22.4)	
BMI^a^ at age 25	21.8 (2.7)		21.9 (2.8)		21.7 (2.6)		21.7 (2.6)		21.8 (2.6)		21.8 (2.6)		21.9 (2.7)		22.0 (2.8)	
Baseline BMI^a^	23.7 (3.4)		24.3 (3.5)		24.0 (3.4)		23.8 (3.3)		23.7 (3.4)		23.6 (3.4)		23.5 (3.4)		23.3 (3.5)	
Overweight (25.0–29.9)		28.2		33.5		32.1		30.2		28.6		27.6		26.1		24.4
Obese (≥30)		4.3		6.9		5.6		5.0		4.4		3.9		3.5		3.4
Waist circumference, cm	78.5 (9.3)		79.1 (9.2)		78.5 (9.0)		78.2 (9.1)		78.3 (9.2)		78.5 (9.3)		78.6 (9.5)		78.6 (9.8)	
Reproductive factors																
Nulliparous		1.3		1.5		1.5		1.4		1.2		1.2		1.1		1.2
No. of live births	2.2 (1.3)		1.8 (1.1)		1.8 (1.2)		1.9 (1.2)		2.1 (1.3)		2.3 (1.3)		2.4 (1.3)		2.6 (1.3)	
≥3 children		30.5		27.5		28.3		29.3		30.3		31.3		31.9		32.4
Oral contraceptive pill used		9.7		10.5		10.3		10.3		10.0		9.7		9.7		9.2
Age at first birth, years^b^	23.4 (3.1)		23.9 (3.3)		23.8 (3.2)		23.5 (3.2)		23.3 (3.1)		23.2 (3.1)		23.1 (3.1)		23.4 (3.0)	
Never breastfed child^b^		2.7		3.7		3.4		3.0		2.7		2.4		2.2		2.5
Breastfeeding per child, months^b^	14.5 (7.5)		13.3 (7.4)		13.2 (7.1)		13.7 (7.1)		14.3 (7.3)		14.7 (7.4)		15.4 (7.7)		16.2 (8.1)	
Premenopause at baseline		45.9		46.1		46.6		47.1		46.4		45.6		45.1		43.9
Age at menopause, years^c^	48.2 (4.3)		47.0 (4.9)		47.6 (4.6)		47.8 (4.4)		48.0 (4.3)		48.3 (4.2)		48.4 (4.2)		48.6 (4.2)	
Reproductive years, years^c^	32.2 (4.6)		35.1 (4.9)		34.6 (4.6)		33.8 (4.4)		33.0 (4.3)		32.3 (4.2)		31.4 (4.2)		29.9 (4.4)	

Abbreviations: BMI, body mass index; MET, metabolic equivalent tasks; SD, standard deviation.

^a^ Weight (kg)/height (m)^2^.

^b^ Among parous women only.

^c^ Among postmenoausal women only.

**Figure 1.**
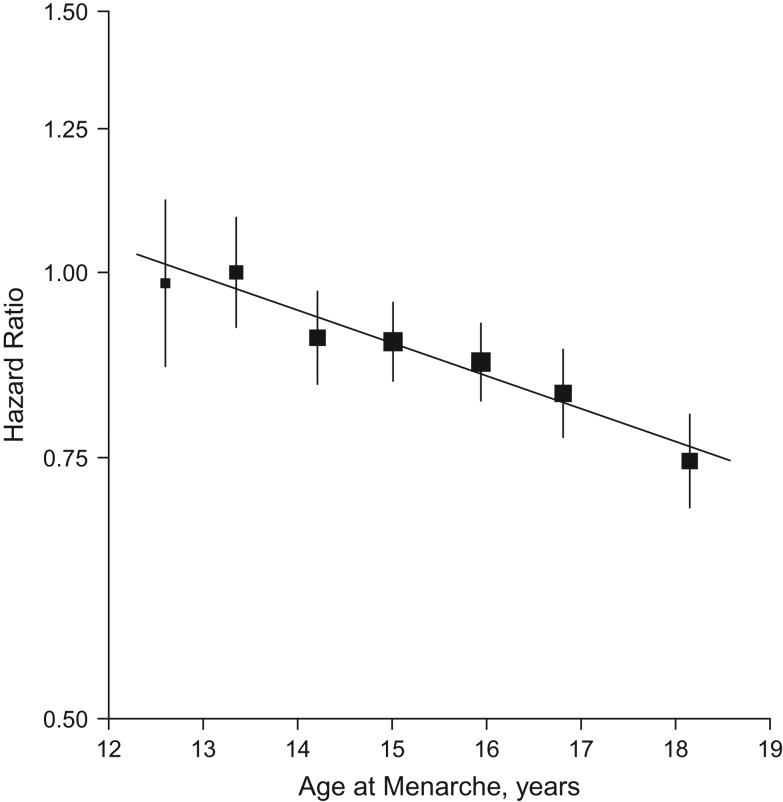
Adjusted hazard ratios for incident diabetes versus self-reported age at menarche (years), China, 2004–2013. Analyses were stratified by age and study area and adjusted for education, household income, smoking status, alcohol intake, blood pressure, physical activity level, menopause status, parity, age at first birth, breastfeeding duration per child, and oral contraceptive use. Women who had menarche at age 13 years were used as the reference category. The hazard ratios are plotted on a floating absolute scale and plotted against the mean usual age at menarche in each category. Squares represent the hazard ratio with area inversely proportional to the variance of the log hazard ratio. Vertical lines indicate the corresponding 95% confidence intervals. The hazard ratio for diabetes was 0.96 (95% confidence interval: 0.94, 0.97) for each year of delay in age at menarche.

**Figure 2.**
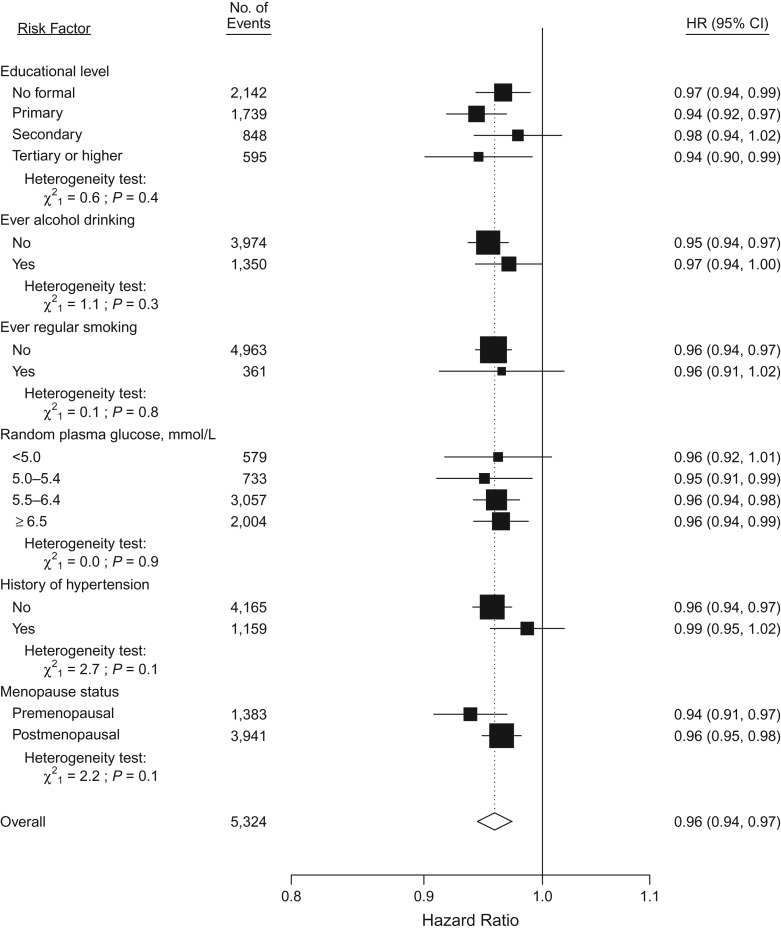
Adjusted hazard ratios (HRs) for incident diabetes associated with each year of delay in age at menarche within various subgroups, China, 2004–2013. Analyses were stratified by age and study area and adjusted for education, household income, smoking, alcohol drinking, blood pressure, physical activity level, menopause status, parity, age at first birth, breastfeeding duration per child, and oral contraceptive use status. Black squares represent the hazard ratios with area inversely proportional to the variance of the log hazard ratio; horizontal lines indicate the corresponding 95% confidence intervals (CIs). The dotted vertical line indicates the overall change of every year delay at age at menarche; the open diamond indicates the overall change and its 95% CI.

**Figure 3.**
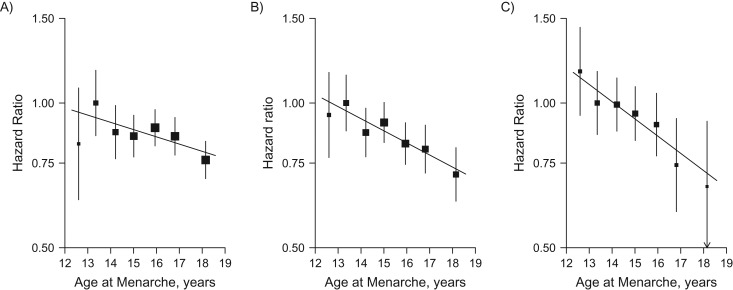
Adjusted hazard ratios for incident diabetes versus age at menarche (years), by birth cohort, China. A) Women born in the 1920s–1940s (*n* = 173,105); B) women born in the 1950s (*n* = 214,502); and C) women born in the 1960s–1970s (*n* = 261,603). Analyses were stratified by age and study area, and adjusted for education, household income, smoking, alcohol drinking, blood pressure, physical activity level, menopause status, parity, age at first birth, breastfeeding duration per child, and oral contraceptive use status. Women who had menarche at age 13 years were used as the reference category. The hazard ratios are plotted on a floating absolute scale and plotted against the mean usual age at menarche in each category. Squares represent the hazard ratios with area inversely proportional to the variance of the log hazard ratio. Vertical lines indicate the corresponding 95% confidence intervals. The corresponding hazard ratios for diabetes for each year delay in age at menarche for each cohort were 0.97 (95% confidence interval: 0.95, 0.99), 0.95 (95% confidence interval: 0.93, 0.98), and 0.93 (95% confidence intervals: 0.90, 0.97), respectively.

**Figure 4.**
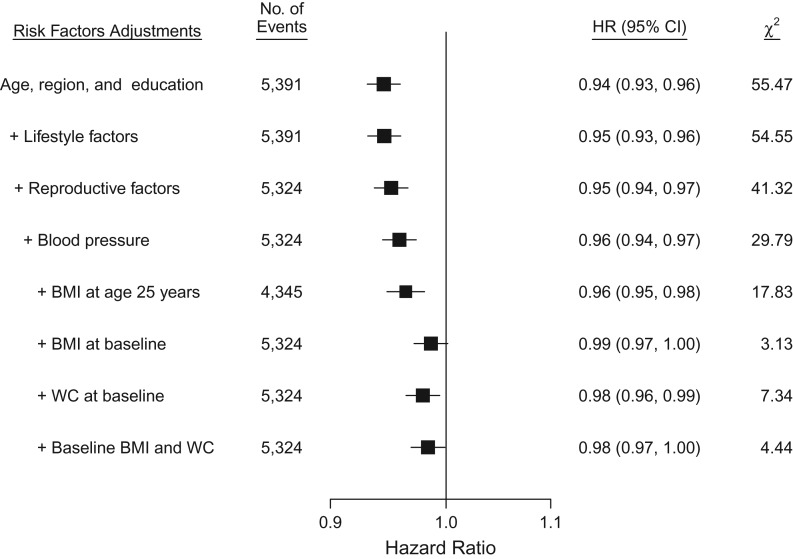
Associations of incident diabetes versus age at menarche (years) before and after adjustment for various risk factors, China, 2004–2013. Each closed square represents a change in hazard ratio of incident diabetes per later age at menarche. BMI, body mass index; CI, confidence interval; HR, hazard ratio; WC, waist circumference.

During 2.0 million person-years of follow-up (mean follow-up duration of 7 years), 5,391 incident cases of diabetes were recorded. After adjustment for socioeconomic, lifestyle and other reproductive factors, age at menarche was inversely associated with risk of incident diabetes, with adjusted hazard ratios of 0.98 (95% confidence interval (CI): 0.86, 1.12), 1.00 (95% CI: 0.92, 1.09), 0.90 (95% CI: 0.84, 0.97), 0.90 (95% CI: 0.84, 0.95), 0.87 (95% CI: 0.82, 0.92), 0.83 (95% CI: 0.77, 0.89), and 0.75 (95% CI: 0.69, 0.80) for those with age at menarche ≤12, 13 (referent), 14, 15, 16, 17 and ≥18 years, respectively (Figure 1). The association was approximately log-linear. For each year of delay of age at menarche, with the same adjustments, the hazard ratio for diabetes was 0.96 (95% CI: 0.94, 0.97) (*P* for trend < 0.001), similar in strength to the subset of women who never smoked, drank or used OCs (hazard ratio (HR) = 0.95, 95% CI: 0.93, 0.97, Web Figure 2). This inverse association was broadly consistent across different population subgroups defined by region, education, alcohol intake, smoking, RBG levels and menopause status (*P* for heterogeneity > 0.50, Figure 2). Across different birth cohorts, the association appeared to be slightly stronger in younger, rather than older generations. The adjusted hazard ratios for diabetes per additional year of age at menarche was 0.93 (95% CI: 0.90, 0.97) for women born in the 1960s–1970s, compared with 0.95 (95% CI: 0.93, 0.98) and 0.97 (95% CI: 0.95, 0.99) for those born in the 1950s and the 1920s–1940s respectively (*P* for trend = 0.05, Figure 3). For each additional year of age at menarche, the hazard ratio for diabetes was 0.94 (95% CI: 0.93, 0.96, χ^2^ = 55.5) after adjustment for age, region and education (model 1), which attenuated to 0.95 (95% CI: 0.94, 0.97, χ^2^ = 41.3) after further adjustment for other lifestyle factors and reproductive factors (model 3), and then to 0.96 (95% CI: 0.94, 0.97, χ^2^ = 29.8) after additional adjustment for measured blood pressure (model 4). Additional adjustment for BMI at age 25 years had little effect on the association (HR = 0.96, 95% CI: 0.95, 0.98; χ^2^ = 17.8). However, additional adjustment for adult adiposity almost completely removed the association, when adjusted for BMI (HR = 0.99, 95% CI: 0.97, 1.00; χ^2^ = 3.1) and for WC (HR = 0.98, 95% CI: 0.96, 0.99; χ^2^ = 7.3) (Figure 4). This substantial reduction in the χ^2^ (from 55.5 to 3.1) suggests that much of the association of age at menarche with diabetes is mediated through its association with adult adiposity, particularly with BMI. The mediating effect of adult adiposity on the association between age at menarche and diabetes appeared to differ between birth cohorts, with BMI adjusted hazard ratios of 0.98 (95% CI: 0.96, 1.00) and 1.00 (95% CI: 0.97, 1.02) for women born after and before the 1950s, respectively.

## DISCUSSION

In the present large study of over 270,000 women from China, there was a log-linear inverse association between age at menarche and risk of diabetes later in life, with a seemingly stronger association in younger rather than older generations. The effects seem largely mediated through adult adiposity, especially among women who were born before the 1950s.

Many previous studies have examined the associations between age at menarche and risk of diabetes, but the results have been largely inconsistent. The discrepancy in findings may be explained in part by differences in study design, sample size, examined populations, age of participants, examined age range of menarche, definition of diabetes, and differences in measurement of adiposity (22). The European Prospective Investigation Into Cancer and Nutrition (EPIC) Study, which included approximately 6,500 incident diabetes cases from 20,000 middle-aged European women, reported that every year of delay in menarche onset was associated with a 9% lower risk of diabetes (8, 10). This association appeared to be largely mediated by adulthood adiposity, with the exception of those with early menarche (8–11 years), for which the increased risk was only partially explained (the adjusted hazard ratio for diabetes decreased from 1.70 to 1.42 after further adjustment for adult BMI) (8). The Shanghai Women’s Health Study, which included 69,385 Chinese women born between 1925–1959, also reported an inverse association between age at menarche and the risk of diabetes (23). This association was completely eliminated after further adjustment for adult BMI, with the hazard ratio decreasing from 0.95 (95% CI: 0.92, 0.98) with adjustment for age and socioeconomic status to 0.99 (95% CI: 0.96, 1.02) after additional adjustment for adult adiposity. However, no other lifestyle or reproductive factors have been adjusted in the analyses, and furthermore, the diabetes status was collected through biennial in-person survey without further validation conducted (23). Together with the present study, these Chinese cohorts extended our findings by showing that the observed inverse associations are even stronger among women with older age at menarche, which could not previously be investigated in Western populations because of the limited number of women who experience menarche at an older age. The Atherosclerosis Risk in Communities (ARIC) Study found that early age at menarche (8–11 years vs. 13 years) was associated with diabetes in white women but not black women, and that further adjustment for adulthood adiposity attenuated these relationships.

Few studies have investigated the generational differences of the association between age at menarche and the risk of diabetes. Two Nurses’ Health Study (NHS) cohorts that included approximately 200,000 American nurses showed an increased risk of diabetes in women with early menarche, with a stronger association in younger (<45 years) than older (≥45 years) women. After adjustment for potential confounders, including BMI at a young age, the relative risks of diabetes for menarche at age 11, 12, 13 (referent), 14, and ≥15 years were 1.18, 1.09, 1.00, 0.92, and 0.95 in the original cohort (aged 34–59 years), and 1.40, 1.13, 1.00, 0.98, and 0.96 in second cohort (aged 26–46 years), respectively. Further adjustment for adult obesity substantially attenuated the associations among older, but not younger generations, which corresponds with what we have found in our cohort of Chinese women (6). This stronger association between age at menarche and diabetes observed in younger generations that cannot be fully explained by increased adult BMI may suggest a potential risk pathway beyond excessive adiposity in younger generations.

The present study suggests that the association between age at menarche and diabetes may be largely mediated by adult adiposity, although the true nature of this relationship is difficult to determine without additional information about childhood adiposity. Several possible pathways have been proposed that reflect a linkage between puberty onset and diabetes. Increasing evidence suggests that high plasma estradiol and testosterone levels and low sex hormone-binding globulin levels may be associated with a higher risk of diabetes in women, independent of adiposity (24). A longer exposure to estrogen induced by earlier menarche may decrease serum sex hormone–binding globulin levels that persist in adulthood (24). Genetic factors may be also involved, as animal studies have shown that overexpression of the RNA-binding proteins Lin28a/b gene exhibits both later pubertal maturation and increased glucose uptake, which provide a possible mechanistic link between early menarche and diabetes risk (22). Early menarche is not only associated with higher prepubertal BMI, but also might lead to the postpubertal accumulation of adipose tissue during development. Both of these prolonged effects of increased obesity may be a main risk factor for diabetes (5, 14). In our cohort of Chinese women, adult adiposity almost completely attenuated the association between age at menarche and diabetes, but adjustment for young adulthood BMI only partially attenuated the association. Lack of information on childhood and adolescent adiposity precluded assessment of the effects of adiposity at an earlier age. However, adiposity in later adulthood is more likely to be a proxy for long-term exposure to being overweight (14), and thus can be more important than BMI in the mediation of increased diabetes risk associated with early menarche earlier in life.

Given the large sample size of both pre- and postmenopausal women, diversity of areas covered, and broadly consistent findings across study population subgroups, the relative risk estimates presented are likely to be generalizable to the population at large. Moreover, the study has several other strengths, including standardized approaches and stringent quality control for data collection, as well as good reproducibility of a comprehensive range of information, which include both lifestyle factors and life-course reproductive factors (4). These allowed us to simultaneously control for potential confounders and thus to reliably assess the association between age at menarche and diabetes.

The study also has limitations. Although we have allowed for a comprehensive range of potential confounders, residual confounding may still exist because of the observational nature of the study. Type 1 diabetes mellitus is associated with delayed menarche, so including women with type 1 diabetes would be expected to have attenuated the association. Although we did not specifically include prevalent and incident type 1 diabetes cases in the analyses, misclassification from other subtype of diabetes among unspecified diabetes (e.g., gestational diabetes mellitus) may still exist. Given that the majority of our participants were aged over 45 years during the follow-up, we believe that the number of cases of any non–type 2 diabetes is very small. The potential recall bias regarding age at menarche is likely to be small because of the high repeatability obtained, that is, the intra-class correlation coefficient of 0.84 between the baseline survey and the resurvey (4).

In summary, we found an inverse association between age at menarche and diabetes, particularly among younger generations, which was largely mediated through increased adiposity associated with early menarche, especially in older generations. Our findings suggest that age at menarche might represent a useful marker to identify women who are at increased risk of developing diabetes in adulthood. Hence, there is a particular need for obesity prevention strategies in girls with early age at menarche. Further studies of the underlying reasons for differences in the patterns of association, as well as the interplay with related generational and other risk factors between Western and Chinese women, are required to fully understand the clinical relevance of these associations.

## Supplementary Material

Web Material

## Abbreviations

BMI: body mass index
CI: confidence interval
HR: hazard ratio
OC: oral contraceptive
RPG: random plasma glucose
WC: waist circumference
