# Supplementary material for: Age at Menarche and Incidence of Diabetes: A Prospective Study of 300,000 Women in China
Source: Am J Epidemiol. 2017 Jun 9;187(2):190–8. doi: 10.1093/aje/kwx219 (PMC5860078; doi:10.1093/aje/kwx219)

Web Table 1. ICD-10 codes for diabetes

ICD-10 codes
E10.0, E10.1, E10.2, E10.3, E10.4, E10.5, E10.6, E10.7, E10.8, E10.9,
E11.0, E11.1, E11.2, E11.3, E11.4, E11.5, E11.6, E11.7, E11.8, E11.9,
E12.0, E12.1, E12.2, E12.3, E12.4, E12.5, E12.6, E12.7, E12.8, E12.9,
E13.0, E13.1, E13.2, E13.3, E13.4, E13.5, E13.6, E13.7, E13.8, E13.9,
E14.0, E14.1, E14.2, E14.3, E14.4, E14.5, E14.6, E14.7, E14.8, E14.9,
E10-E14 (without any decimal)

Web Figure 1. Body mass index (BMI), waist circumference and random blood glucose versus age at menarche, with adjustment for age, region, education, household income, smoking, alcohol drinking, blood pressure and physical activity

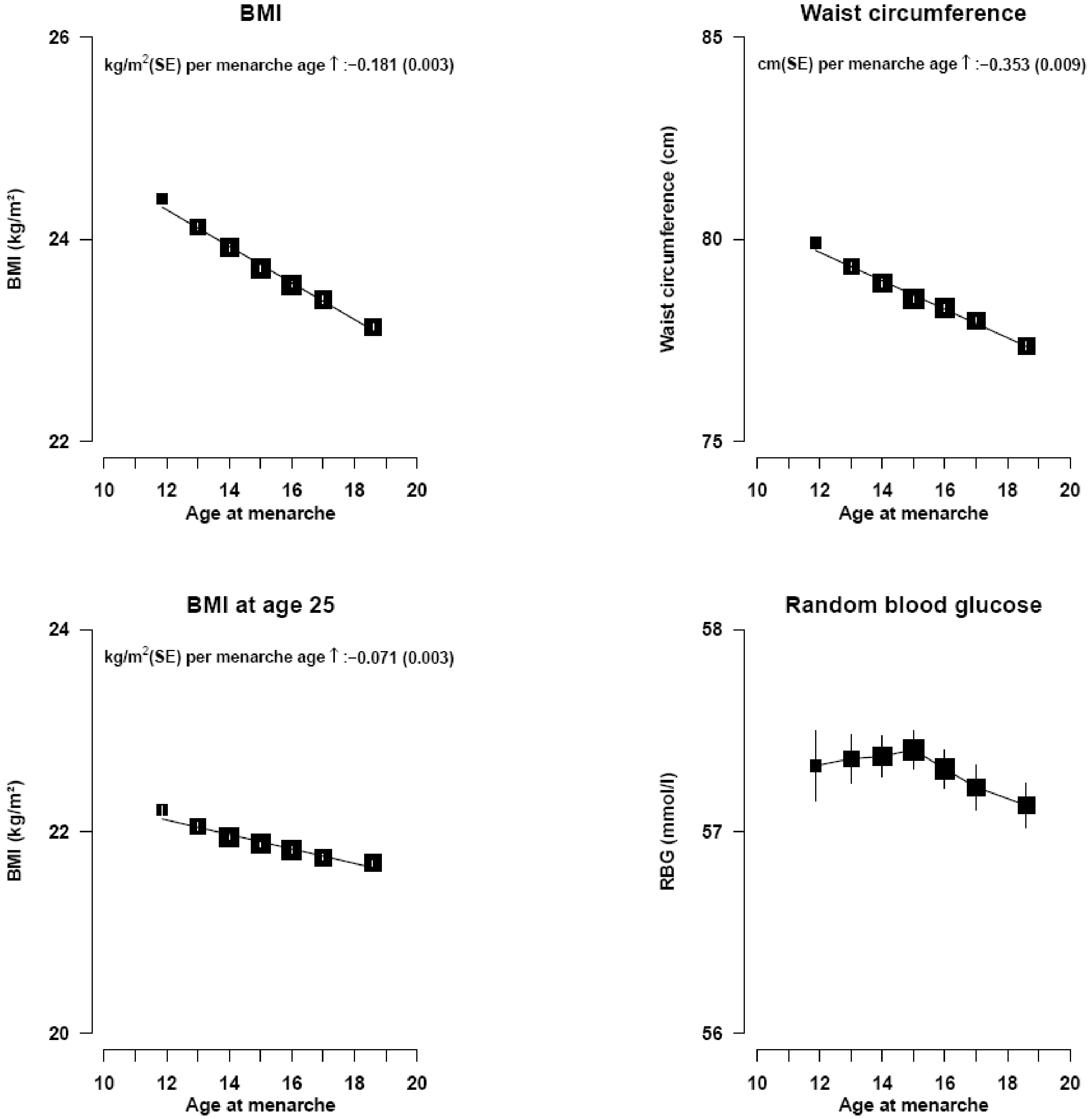

Web Figure 2. Adjusted hazard ratios for incident diabetes versus age at menarche (years) among women who never smoked or consumed alcohol, and who never used OCs, adjusted by age, region, lifestyle risk factors and reproductive factors

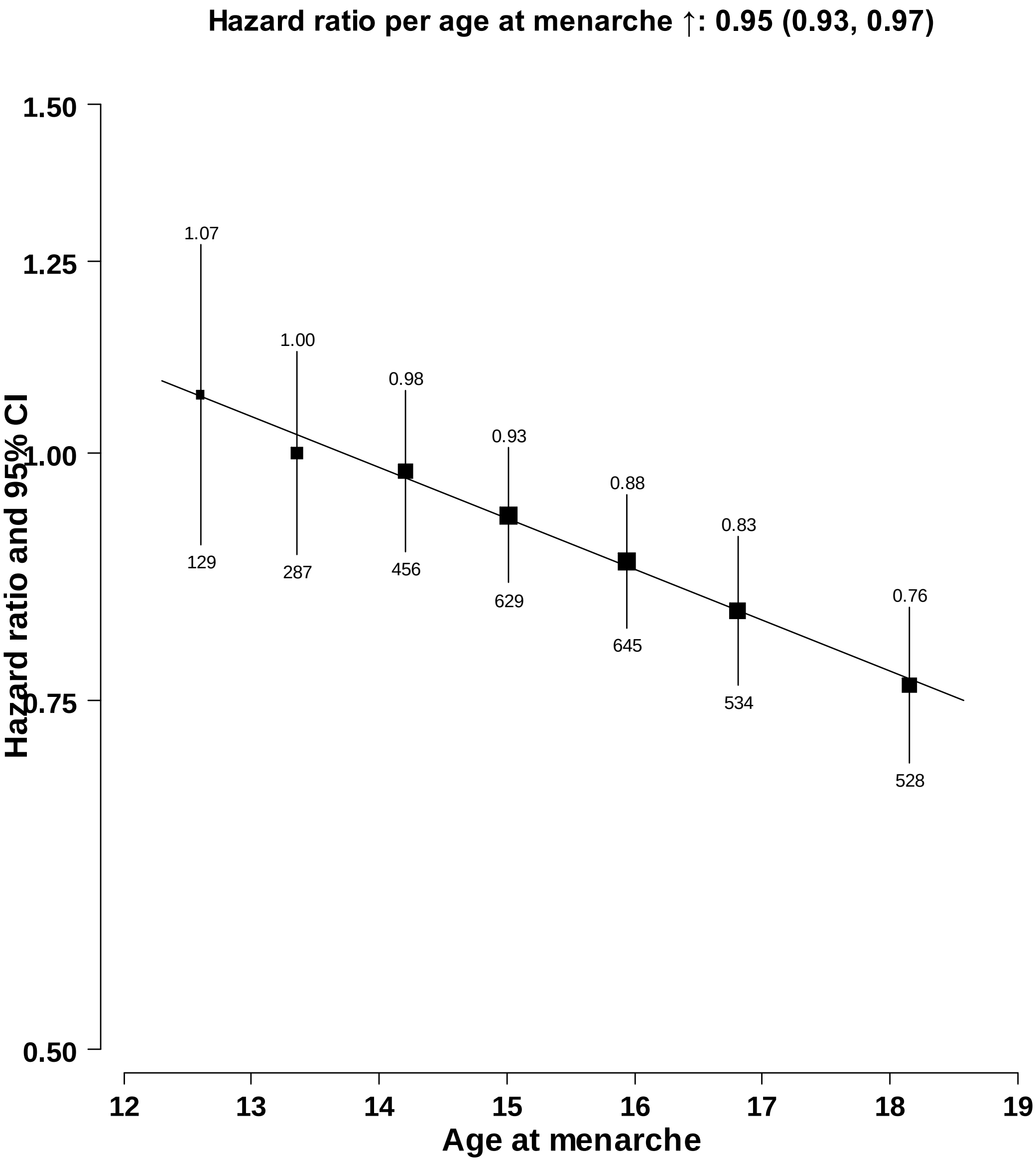

Supplement: Web Material [file kwx219yang_web_materialfinal.pdf]
